# Serum Apo A-1 and Its Role as a Biomarker of Coronary Artery Disease

**DOI:** 10.7759/cureus.941

**Published:** 2016-12-24

**Authors:** Salma Rahim, Hafez Mohammad A Abdullah, Yousaf Ali, Uzma I Khan, Waqas Ullah, Muhammad A Shahzad, Muhammad Waleed

**Affiliations:** 1 Biochemistry, Bacha Khan Medical College; 2 Internal Medicine, Khyber Teaching Hospital; 3 Public Health, Aga Khan University; 4 Internal Medicine, University of Arizona; 5 Internal Medicine, Hayatabad Medical Complex

**Keywords:** apo a-1, coronary artery disease, hdl

## Abstract

**Objectives:**

To evaluate the role of apolipoprotein(Apo A-1) as a biomarker of coronary artery disease (CAD) and its comparison with the traditional marker high-density lipoprotein (HDL).

**Methodology:**

One hundred patients proven to have coronary artery disease by angiography were recruited and their serum biomarkers were compared with 100 normal individuals adjusted for age and sex.

**Result:**

The mean +/-standard deviation (SD) value of plasma Apo A-1 levels in the normal individuals were observed to be 207.42 +/- 41.35 (mg/dL) against 90.69 +/- 20.77 (mg/dL) in the cardiac patients. On the other hand the serum HDL levels were 52.93 +/-33.58 (mg/dL) in the normal individuals and 37.86 +/- 23.19 (mg/dL) in the cardiac patients. Both of these differences were statistically significant (p < 0.001). For Apo A-1, a large proportion of patients (85%) were found to be in the abnormal range when compared to the control group in which only 7% had an abnormal value. For HDL, a majority (70%) of the cardiac patients had abnormal values while 40% of the normal individuals also had abnormal values. The sensitivity of Apo A-1 for detecting CAD was 85%, while for HDL, it was only 69%. Similarly, the specificity of Apo A-1 for detecting CAD was 93%, while for HDL, it was 60%. When plotted on a receiver operating characteristic (ROC) curve, Apo A-1 had a much larger area under the curve when compared to HDL.

**Conclusion:**

This study suggests that Apo A-1 may, in fact, be more sensitive than HDL as a predictor of CAD. However, to completely elucidate its role as a biomarker, to set target serum levels and to increase its clinical use, further studies are required.

## Introduction

Plasma cholesterol levels are important predictors of coronary artery disease (CAD) [1-2]. Various plasma cholesterol parameters like HDL, LDL levels, and indices like total cholesterol/HDL and LDL/HDL have been used as predictors of CAD [3-4]. It has been observed that plasma Apo A-1 levels, the major protein of HDL, may also be a reliable marker of CAD [5-7]. Many studies have suggested that plasma Apo A-1 and apolipoprotein (Apo B) levels may, in fact, be more accurate predictors of CAD [8-11], especially in those patients having low or normal LDL cholesterol levels [12]. In fact, some studies suggest that the ratio of plasma Apo B to plasma Apo A-1 may be the most accurate predictor for CAD [9-12]. However, there are still some limitations to the usage of Apo A-1 or the Apo B/Apo A-1 ratio and it still remains to be seen what additional information plasma Apo A-1 levels can provide that plasma HDL levels cannot [9].

Coronary artery disease is reported to be the leading cause of morbidity and mortality worldwide and as such it is an important public health concern [13-14]. Early diagnosis and treatment can prevent many cases of acute coronary syndrome. Besides its mortality benefit, a good screening test for coronary artery disease can significantly reduce the human and financial resources dedicated to the management of coronary artery disease related morbidity and mortality. Studies have pointed to a wide spectrum of genetic and acquired risk factors associated with CAD [14]. However, whatever the risk factors are, there is usually an underlying defect in cholesterol homeostasis that leads to coronary artery atherosclerosis, which eventually leads to coronary artery disease. The most important factor is the balance between the pro-atherogenic factors low-density lipoprotein (LDL) and Apo B and the anti-atherogenic factors HDL and Apo A-1 levels. LDL, along with its major component Apo B, is tasked with taking hepatic cholesterol to tissues to fulfill their needs, and HDL and Apo A-1 bring back any excess cholesterol deposited in the blood vessels to the liver for excretion.

Apo A-1 is the major protein component of high-density lipoprotein cholesterol (HDL-C). It is also initially a part of chylomicrons that are released by enterocytes, but it is then transferred back onto HDL-C. It has a molecular weight of 28.1 kDa and is encoded by the apolipoprotein A1 (Apo A-1 ) gene. It plays an important role in cholesterol metabolism, along with HDL-C. The main function of HDL-C is to take up cholesterol in tissues and direct it back to the liver for excretion through the bile. Cholesterol cannot be metabolized and used as a source of energy in the human body, so excretion through bile is the only way the body can get rid of excess cholesterol after the body's requirements have been met. Apo A-1 helps HDL in this task. Apo A-1 also activates lecithin-cholesterol acyltransferase (LCAT), the enzyme present on HDL that esterifies the cholesterol picked up by HDL and, hence, renders it lipid-soluble so that it can be sequestered deep within the HDL particles, ensuring that the HDL particles do not lose the cholesterol esters again. The sequestered cholesterol esters can then be taken up by the liver, along with the HDL particles. Apo A-1 has several other important functions as well and its deficiency and mutations have been implicated in various diseases ranging from a hypercoagulable state to Alzheimer's disease and amyloidosis [15-16]. Apo A-1 is reported to have a stabilizing effect on prostacyclin and may have an anti-clotting role to play [17]. A recent study suggested Apo A-1 has anti-inflammatory and anti-tumorigenic effects, in addition to its cardioprotective effects [18]. It also has a negative correlation with insulin resistance [19]. HDL deficiency states may occur due to defects in the Apo A-1 protein, including Tangier's disease. Polymorphisms in the Apo A-1 gene have led to the discovery of conditions with a greatly increased risk of developing early atherosclerosis and CAD [15]. However, in some cases, the polymorphic forms may be protective against CAD.

The serum level of HDL has traditionally been considered to be an important biomarker of CAD; however, recent studies have indicated that in some cases higher HDL levels may not always be protective [20]. One interesting observation mentioned by a meta-analysis of eight statin trials was that a rise in HDL after statin therapy had no significant cardiovascular benefit, whereas a rise in Apo A-1 resulted in a significant reduction of cardiovascular events [21]. This has challenged the long-held concept that high HDL levels always reduce the risk of CAD. Also, the serum lipid and cholesterol levels have been unable to explain the prevalence of CAD in patients with a normal lipid profile [22]. Considering the research already mentioned indicating the superiority of Apo A-1 and Apo B as biomarkers, it is now genuinely thought that these proteins can replace HDL and LDL as the primary markers of CAD. Various studies have already indicated that low Apo A-1 is an independent risk factor for CAD [23]. It is reported also to have a beneficial effect on non-ischemic heart failure through its anti-inflammatory effect [24]. It is also reported to be an early marker of CAD and may be helpful in the screening of younger patients [25]. Apo A-1 is also suggested to be a marker of the severity of coronary artery obstruction [26]. This study was carried out to find the correlation between CAD and serum Apo A-1 and its utility as a biomarker of CAD as compared to HDL. Informed consent was obtained from all patients for this study.

## Materials and methods

This research was conducted at the Postgraduate Medical Institute (PGMI) Lady Reading Hospital (LRH) Peshawar, after the approval of the Ethics Committee of PGMI. Two hundred subjects were recruited. One hundred of them were cardiac patients who underwent angiography and were confirmed to be having CAD. The remaining 100 were individuals with no atherosclerosis on angiography. The subjects were adjusted for age, and various parameters and serum biomarkers, including the fasting lipid profile, Apo A-1, Apo B, serum leptin, and serum glucose levels, were measured. The lipid profile was determined by the calorimetric method using a kit provided by Roche, Switzerland. Apo A-1 and Apo B were determined by the immunoturbidimetric method utilizing kits supplied by Roche, Switzerland.

## Results

A total of 200 (n = 200) subjects were included in the study. One hundred were CAD patients, proven by angiography to have significant atherosclerosis and placed in the patient group, while the other 100 subjects were individuals of a similar age group, sex, race, geographic location, and socioeconomic status with no atherosclerosis on angiography placed in the control group. Various parameters were measured and expressed as mean+SD. Then the sensitivity, specificity, positive predictive values, negative predictive values, and ROC curves for Apo A-1 and HDL were derived.

The biochemical characteristics of the cardiac patients and the control group are summarized in Table-1. It shows that there is a very significant difference in serum Apo A-1 levels in the cardiac patients and the normal healthy individuals. The mean+SD of plasma Apo A-1 levels in the normal individuals was observed to be 207.42 +/- 41.35 (mg/dL), against 90.69 +/- 20.77 (mg/dL), in the cardiac patients. On the other hand, the mean +/- SD serum HDL levels were 52.93 +/- 33.58 (mg/dL) in the healthy individuals and 37.86 +/- 23.19 (mg/dL) in the cardiac patients. Both of these results show a statistically significant (p < 0.01) difference between the levels of both Apo A-1 and HDL in coronary artery disease patients when compared with the healthy individuals (Table 1).

**Table 1:** Biochemical characteristics of cardiac patients and controls

	Mean	SD +/-
Mean Apo A-1 level in Diseased	90.69mg/dl	20.77668mg/dl
Mean Apo A-1 level in Normal	202.2mg/dl	50.64812mg/dl
Mean HDL level in Diseased	35.448mg/dl	16.81668mg/dl
Mean HDL level in Normal	41.50mg/dl	9.338324mg/dl

Apo A-1 shows higher sensitivity and specificity than HDL. The sensitivity and specificity of Apo A-1 in detecting diseased cases was calculated to be 85% and 93%, respectively, as compared to that of HDL in which case the sensitivity and specificity were 69% and 60%, respectively. The positive and negative predictive values of Apo A-1 were 92% and 86%, respectively, as compared to that of HDL which is 63% and 66%, respectively (Tables 2-3).

**Table 2:** 2x2 table for Apo A-1 and sensitivity, specificity, positive predictive value and negative predictive value for Apo A-1

2x2 table for Apo A-1			
	Diseased	Not Diseased	Total
Positive	85	7	92
Negative	15	93	108
Total	100	100	200
Sensitivity		85%	
Specificity		93%	
Positive predictive value		92.3913	
Negative predictive value		86.1111

**Table 3:** 2x2 table for HDL and sensitivity, specificity, positive predictive value and negative predictive value for HDL

2x2 table for HDL			
	Diseased	Not Diseased	Total
Positive	69	40	109
Negative	31	60	91
Total	100	100	200
Sensitivity		69	
Specificity		60	
Positive predictive		63.30275	
Negative predictive		65.93407	

**Figure 1:**
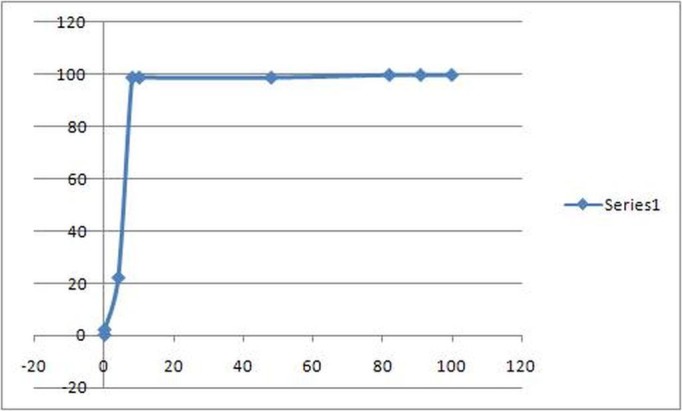
ROC curve for Apo A1

**Figure 2:**
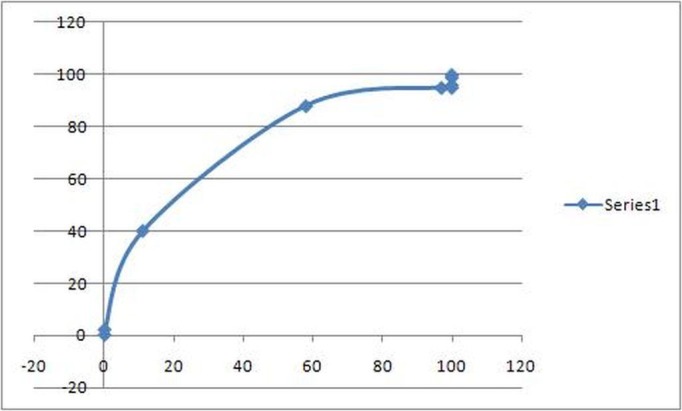
ROC curve for HDL

ROC curve analysis of both the tests also showed a significant difference. In the case of Apo A-1 the area under the curve as determined by its ROC curve was 0.94 while in the case of HDL the area under the curve of ROC curve turned out to be 0.70 (Table 4) (Figures 1-2).

**Table 4:** ROC curves for Apo A-1 and HDL

ROC Curve			
Apo A-1			
Cut off Value	False +	True +	AUC
0	0	0	0
40	0	2	0.0048
80	4	22	0.0242
120	8	99	0.0198
160	10	99	0.3762
200	48	99	0.3383
240	82	100	0.09
280	91	100	0.09
320	100	100	
		Area under the curve	0.9433
ROC Curve			
HDL			
Cut off Value	False +	True +	AUC
0	0	0	0
15	0	2	0.0231
30	11	40	0.3008
45	58	88	0.35685
60	97	95	0.0285
75	100	95	0
90	100	96	0
105	100	99	0
120	100	100	
		Area under the curve	0.70925

Though Apo A-1 gave us higher sensitivity and specificity as compared to HDL, we can also take the advantage of combined screening with multiple tests. In our study simultaneous screening with Apo A-1 and HDL increased the sensitivity level to 96% (Table 5). Specificity was also increased to 96% by multi-stage screening with Apo A-1 followed by HDL (Table 6).

**Table 5:** Simultaneous screening with Apo A-1 and HDL

Simultaneous screening with Apo A-1 and HDL			
2x2 table for Apo A-1			
	Diseased	Not Diseased	Total
Positive	96	43	139
Negative	4	57	61
Total	100	100	200
		Sensitivity	96
		Specificity	57

**Table 6:** Multi-stage screening with Apo A-1 and HDL

Multi stage screening with Apo A-1 and HDL			
2x2 table for Apo A-1			
	Diseased	Not Diseased	Total
Positive	58	4	62
Negative	42	96	138
Total	100	100	200
		Sensitivity	58
		Specificity	96

## Discussion

The results of our study showed a significant difference in the serum Apo A-1 levels of the coronary artery disease patients and normal controls. It showed that the serum Apo A-1 levels in normal healthy individuals was mean +/- SD 207.42 +/- 41.35 (mg/dL) and in cardiac patients was 90.69 +/- 20.77 (mg/dL) which is a very significant difference. A very large majority of healthy individuals (93%) had their serum Apo A-1 levels within the normal range while most of the cardiac patients (85%) had an abnormal serum Apo A-1 level. On the other hand the mean +/-SD serum HDL levels were 52.93 +/- 33.58 (mg/dL) in healthy individuals and 37.86 +/- 23.19 (mg/dL) in cardiac patients. Statistically, this difference is also significant. However, only 60% of the healthy individuals had HDL levels within the normal range and only 70% of the cardiac patients had an abnormal HDL level. Apo A-1 had a much higher sensitivity and specificity for detecting coronary artery atherosclerosis when compared with serum HDL levels. When plotted on ROC curve, Apo A-1 had a much higher area under the curve when compared with HDL. The ROC curve of Apo A-1 showed the area under the curve to be 0.94 as against 0.70 for HDL, which suggested Apo A-1 as a more sensitive diagnostic test. Combining the two tests for better results in multi-stage screening did not add any significant amount of sensitivity; however, simultaneous screening with the two tests increased the sensitivity up to 96%. These findings suggest that the serum Apo A-1 may be a more sensitive marker of coronary artery disease. In coronary artery disease patients, our study showed that Apo A-1 levels serve as a better diagnostic tool than HDL level as depicted by the higher sensitivity and specificity of Apo A-1 and a higher area under the curve on the ROC curve as compared to HDL.

## Conclusions

Our study is consistent with many previous studies [12, 27-29], which, as discussed earlier, suggest that serum Apo A-1 may actually be more sensitive in predicting coronary artery disease as compared to serum HDL. Apo A-1 is also an early marker of CAD and is useful in estimating the risk of CAD in younger patients. Its use as a clinical tool should be increased for the better management and prevention of coronary artery disease. Its higher sensitivity may prove to reduce morbidity and mortality in the long run and, consequently, also reduce the financial burden posed by this disease. It is also more convenient to use as non-fasting samples are required as opposed to serum cholesterol levels.

The relatively smaller sample size of our study may have affected the degree of correlation of CAD with Apo A-1 and HDL due to a few outliers. Prospective studies with large cohorts are required to completely elucidate the correlation between serum Apo A-1 levels and CAD, to set the guidelines for the target plasma values, and increase its clinical use in the prevention of CAD.
